# Supplementary material for: HSP70/IL-2 Treated NK Cells Effectively Cross the Blood Brain Barrier and Target Tumor Cells in a Rat Model of Induced Glioblastoma Multiforme (GBM)
Source: Int J Mol Sci. 2020 Mar 25;21(7):2263. doi: 10.3390/ijms21072263 (PMC7178276; doi:10.3390/ijms21072263)
Supplement: Supplementary file 1 [file ijms-21-02263-s001.pdf]

Supplementary Figure 1

A

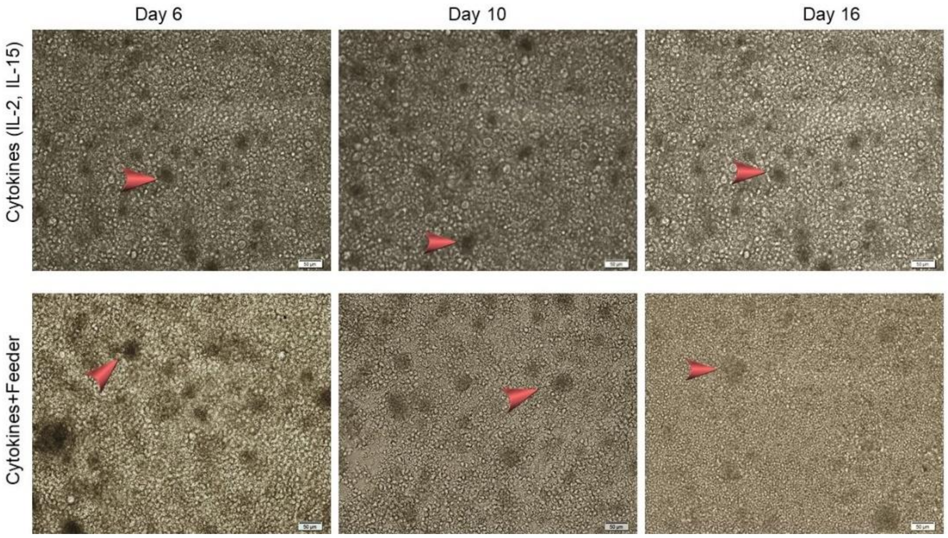

B

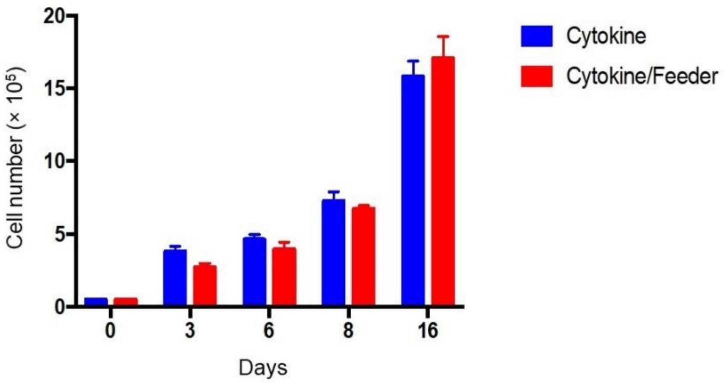

Figure S1: Isolation, characterization and proliferation of NK cells. **A)** The morphology of NK cells after 6, 10 and 16 days of incubation in cytokine-enriched (IL-2/IL-15) medium (top row), or cytokine/feeder-enriched (IL-2/IL-15/K562 cells) medium (bottom row). Red arrows show the colonies of expanded NK cells. **B)** The growth rate comparison of expanded NK cells in the presence of cytokines only (IL-2/IL-15) or cytokines and feeder (IL-2/IL-15/K562 cells) over time. No significant differences between the two groups were observed (*p*-values>0.1).

Supplementary Figure 2

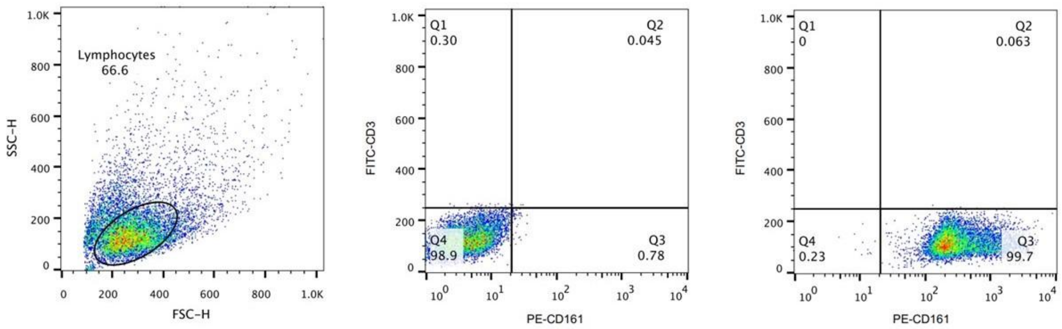

Figure S2. The frequency of CD3-CD161 positive NK cells in a splenocyte-derived mononuclear cell population, after FACS with more than 99% purity.

Supplementary Figure 3

A

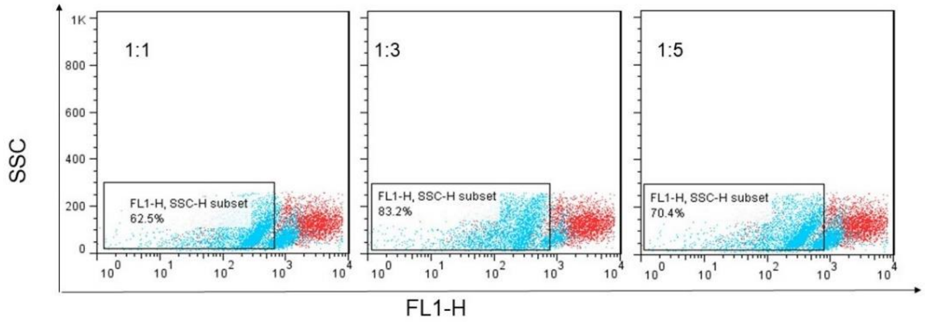

B

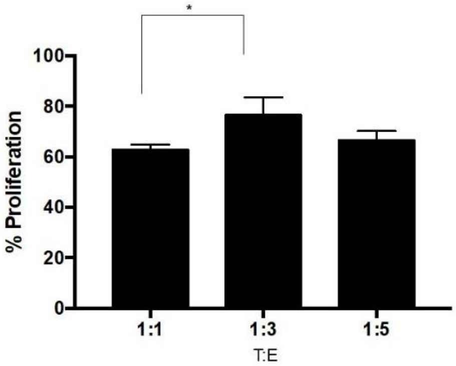

Figure S3: Functional assessment of NK cells. **A)** Flow cytometry based results of CFSE-labeled NK cells in different concentrations, which shows proliferation in co-culture assays with different ratios of C6 cells (T:E 1:1; 1:3; 1:5), highlighting the augmented proliferation rate at a T:E ratio of 1:3. **B)** Quantitative comparison of different initial quantities of CFSE-NK cells on proliferation, in co-culture with a constant number of C6 cells. All data presented as mean±SD of 3 independent experiments (*p*-value 1:1 *vs.* 1:3: 0.0280; 1:1 *vs.* 1:5: 0.6208; 1:3 *vs.* 1:5: 0.0920).

Supplementary Figure 4

A

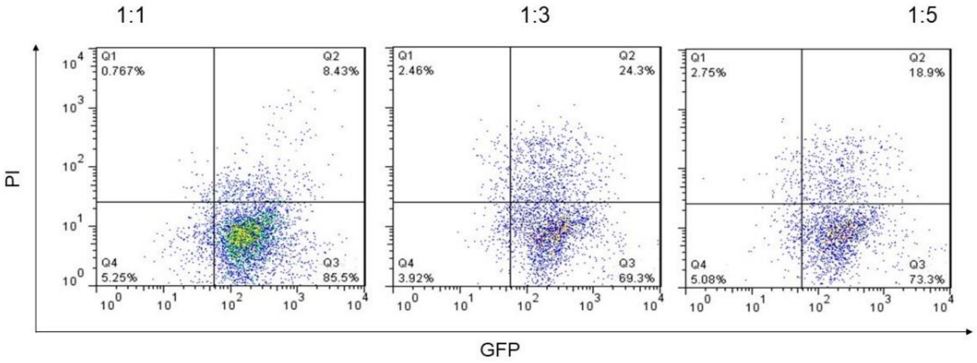

B

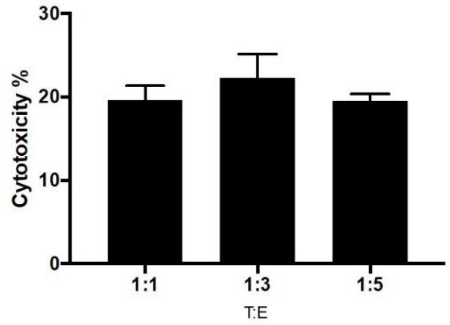

C

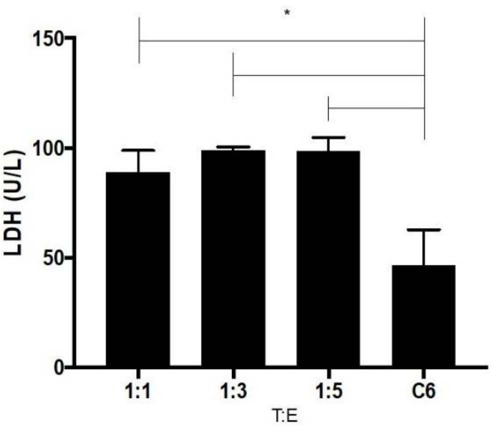

Figure S4: Cytotoxicity assay of expanded NK cells at different E:T ratios. **A)** Representative dot plots show the extent of cytotoxicity of different concentrations of NK cells on GFP-labeled C6 cells (T: E, 1:1; 1:3; 1:5). As dead cells were labeled with PI, GFP$^+$/PI$^+$ cells are indicative of tumor cell death. The percentage of C6 cell death was analyzed by flow cytometry. **B)** Quantitative

analysis of the cytotoxic effects of different concentrations of NK cells on a constant number of GFP-labeled GBM C6 cells. Although an apparent increase in cytotoxicity could be observed when cells were studied in a 1:3 ratio, no significant differences were detected between the different ratios applied. **C)** Graph representing supernatant levels of LDH, a cell death biomarker, vs C6 cells as negative controls, *p-value<0.05 compared to C6; 1:1 (Adjusted *p*-value: 0.0444); 1:3 (Adjusted *p*-value: 0.0218), 1:5 (Adjusted *p*-value: 0.0225) *vs.* negative control.
